# The impact of ageing on the distribution of preformed anti-HLA and anti-MICA antibody specificities in recipients from eastern China prior to initial HSCT

**DOI:** 10.1186/s12979-024-00417-4

**Published:** 2024-02-20

**Authors:** Qinqin Pan, Xiao Ma, Yajie You, Yuejiao Yu, Su Fan, Xiaoyan Wang, Mengyuan Wang, Ming Gao, Guangming Gong, Kourong Miao, Jie Shen, Xiaoyu Zhou

**Affiliations:** 1 HLA Lab, Department of Transfusion, The First Affiliated Hospital of Nanjing Medical University, Jiangsu Province Hospital, Nanjing, 210029 China; 2 Department of Pharmacy, Jinling Hospital, Nanjing University School of Medicine, Nanjing, 210002 China; 3 Department of Hematology, The First Affiliated Hospital of Nanjing Medical University, Jiangsu Province Hospital, Nanjing, 210029 China

**Keywords:** Ageing, Anti-HLA antibody specificities, Anti-MICA antibody specificities, HSCT, East China

## Abstract

**Background:**

With the development of Hematopoietic Stem Cell Transplantation (HSCT) technology, increasing numbers of elderly patients were undergoing allogeneic HSCT and elderly patients with hematologic malignancies could benefit most from it. Preformed donor-specific human leukocyte antigen (HLA) antibodies (DSA) were associated with graft failure in HLA-mismatched allogeneic HSCT and the absence of DSA was the main criterion of selecting the donor. Except for sensitization events such as transfusion, pregnancy or previous transplantation, ageing affects the humoral immune response both quantitatively and qualitatively. To evaluate the prevalence and distribution of anti-HLA and antibodies of MHC class I chain related antigens A (MICA) specificities in different age groups before initial HSCT would provide HLA and MICA specific antibody profiles under the impact of ageing, which could provide meaningful information in the process of selecting suitable HLA-mismatched donors by avoiding preformed DSA.

**Results:**

There were no significant differences in the distribution of anti-HLA class I, class II and anti-MICA antibodies among the three age groups in this study except that a significant lower negative ratio of anti-HLA class I, class II antibodies and higher positive rate of MICA antibodies with maximum mean fluorescent intensity (MFI) > 5000 in the elderly than in young age group. The distribution of antibody specificities against HLA -A, -B, -C, -DR, -DQ, -DP and MICA antigens in the three age groups were generally consistent. The anti-HLA class I antibody specificities with higher frequencies were A80,A68;B76,B45;Cw17, which were unlikely to become DSA in Chinese. Anti-HLA class II antibody specificities were more likely to become potential DSA than class I.DR7, DR9, DQ7, DQ8 and DQ9 were most likely to become potential DSA.

**Conclusions:**

The prevalence of anti-HLA and anti-MICA antibodies increased slightly as age increased. While ageing had a small impact on the distribution of antibody specificity frequencies against HLA-A, -B, -C, -DR,-DQ, -DP and MICA antigens in recipients awaiting initial HSCT from East China. The risk of developing preformed DSA was basically consistent in the three age groups and the elderly group might be more favorable in HLA-mismatched HSCT due to higher positive rate of anti-MICA antibody.

## Background

Allogeneic hematopoietic stem cell transplantation (HSCT) is recognized as an effective therapy for the majority of malignant hematologic diseases. Human leukocyte antigen (HLA) mismatched donors are now increasingly considered for transplantation in the absence of HLA-matched donors due to improved transplant outcomes. The causes of graft failure in HLA-mismatched HSCT were multifactorial and anti-HLA antibodies were affirmed to increase the risk of graft failure. Significantly, preformed donor-specific anti-HLA antibodies (DSA) activated the complement cascade and caused destruction of the donor cells resulting in allograft rejection by targeting donor anti-HLA antigens present on the surface of hematopoietic progenitor cells, which played as an important risk factor of graft failure in HLA-mismatched HSCT [1, 2]. The absence of DSA in the recipient sera was the main criterion of selecting the donor [3].Pregnancy, prior transplantation and blood transfusion represented the major risk factors of developing preformed anti-HLA antibodies [4]. Although HLA sensitization resulting from transfusion is less robust and generally shorter lived than that resulting from transplantation [5]. While blood transfusion was a common source of sensitization for patients prior to HSCT, due to the effects of chemotherapeutic treatments for hematologic malignancies.

The immune system gradually decreases in individuals over 50 years old, that is immunosenescence, which is characterized by diminished immune response [6, 7]. After the age of 50 years old, the bone marrow is gradually replaced by adipose tissue and becomes yellow bone marrow. Constant exposure to new and persisting antigens and the need to replace cellular attrition with newly built cells lead to profound remodeling of the immune system after the age of 50 years. Ageing is accompanied by change in humoral immune function for the changes in all B cell compartments and naive B cells are replaced by antigen-experienced memory cells, which may result in changes of antibodies [8].

With the development of HSCT technology, increasing numbers of elderly patients with hematologic malignancies are undergoing allogeneic HSCT, with more emphasis on comorbidities than age alone for patient selection [9]. Besides, for example acute myeloid leukemia (AML) in older patients tends to harbor less favorable cytogenetic and molecular patterns than in younger patients, resulting in a higher risk for chemoresistance and relapse [10]. Therefore, elderly patients could benefit most from the immunotherapeutic potential of allogeneic transplant. In addition, antibodies of MHC class I chain related antigens A (MICA) had been educated much interest in HSCT, which showed that the presence of anti-MICA antibody before transplantation could confer protection against graft-versus-host disease (GVHD) [11]. Single antigen flow bead (SAFB) assay could identify antigenic specificity of anti-HLA and anti-MICA antibodies with the strength interpreted using mean fluorescent intensity (MFI) values [12].

However, there was a lack of prevalence research about preformed anti-HLA and anti-MICA antibodies in recipients prior to initial HSCT associated with ageing. To evaluate the prevalence and frequencies of anti-HLA and anti-MICA specificities in different age groups before initial HSCT would provide HLA and MICA specific antibody profiles under the influence of ageing, which could help confirm the common HLA specificity and potential DSA in recipients of different age groups and provide meaningful information in the process of selecting suitable HLA-mismatched donors by avoiding preformed DSA.

## Results

### Patient characteristics

In this study, 426 patients awaiting HSCT were retrospectively analyzed for the prevalence of anti-HLA and anti-MICA antibodies. All the patients were for initial HSCT and with no history of organ transplantation.The patient characteristics were outlined in Table 1. There were no significant differences in transfusion, pregnancy, gender ratio among the three age groups (*P* > *0.05*).

**Table 1:** The patient characteristics

	**Group**	**Transfusion**	**Pregnancy**	**Patients (*n* = 426)**
**Age**	≤ 30 years	76(62.3%)	31(25.4%)	Male 74(17.4%)
				Female 48(11.3%)
	31–50 years	118(67.0%)	72(40.9%)	Male78 (18.3%)
				Female 98(23.0%)
	≥ 51 years	98(76.6%)	53(41.4%)	Male 75 (17.6%)
				Female 53(12.4%)
**Diagnosis**	AML(Acute myeloid leukemia)	121(28.4%)		
	ALL(Acute lymphoblastic leukemia)	72(16.9%)		
	MDS(Myelodysplastic syndromes)	67(15.7%)		
	CMML(Chronic myelomonocytic leukemia)	52(12.2%)		
	AA (Aplastic anemia)	35(8.2%)		
	Others	79(18.5%)		

### Distribution of anti-HLA and anti-MICA antibodies in recipients of different age groups

The distribution of anti-HLA class I, class II and anti-MICA antibodies in the three age groups were shown in Table 2. The positivity rates of anti-HLA and anti-MICA antibodies increased slightly as age increased. As for anti-HLA class I antibodies, there were no significant differences at negative ratio, positive rate of maximum MFI 500–3000, 3000–5000, > 5000(including MFI 5000–10000 and > 10,000) among the three age groups except that a significant lower negative ratio in the elderly than in the young group (*P* = 0.0012). As for anti-HLA class II antibodies, there were no significant differences at the negative ratio, positive rate of maximum MFI 500–3000, 3000–5000, > 5000 among the three age groups, except that a significant lower negative ratio in the elderly than in young group (*P* = 0.0037). As for anti-MICA antibodies, there were no significant differences at the negative ratio, positive rate of maximum MFI 500–3000, 3000–5000, > 5000 among the three age groups except that a significant higher positive rate of maximum MFI > 5000 in the elderly than in young group (*P* = 0.0027). In the young group, the negative ratio of anti-HLA class I antibodies was significantly lower than class II (*P* = 0.037), and class II than anti-MICA (*P* = 2.71538E-11). In the middle and elderly group, there were no significant differences in the negative ratio between anti-HLA class I and class II, but both significant lower than anti-MICA (*P* < 0.05).

**Table 2:** The distribution of anti-HLA class I, class II and anti-MICA antibodies in the three age groups

Antibody	MFI	Distribution (%)		
		**Young**	**Middle**	**Elderly**
**Anti-HLA class I antibody**	Negative	33.6	28.4	19.5
	500–3000	36.9	33.5	44.5
	3000–5000	10.7	14.2	14.8
	5000–10000	9.8	13.1	9.4
	> 10,000	9	10.8	11.7
**Anti-HLA class II antibody**	Negative	46.7	36.9	28.9
	500–3000	39.3	40.9	47.7
	3000–5000	4.1	6.3	9.4
	5000–10000	5.7	6.8	6.3
	> 10,000	4.1	9.1	7.8
**Anti-MICA antibody**	Negative	86.9	84.7	78.1
	500–3000	11.5	11.9	14.8
	3000–5000	1.6	1.7	3.1
	5000–10000	0	1.7	3.9
	> 10,000	0	0	0

### Distribution of antibody specificities against HLA-A,-B,-C,-DR,-DQ,-DP and MICA antigens in recipients of different age groups

The antibody specificity frequencies against HLA-A, -B, -C,-DR,-DQ,-DP and MICA antigens in three age groups were shown in Tables 3, 4, 5, 6, 7, 8 and 9. On the whole, the distribution of specificities against HLA-A, -B, -C, -DR,-DQ, -DP and MICA antigens were consistent among the three age groups. The top two frequent anti-HLA-A specificities in the three age groups were A80(A*80:01) and A68(A*68:02) and the corresponding antigen frequencies were very low in Chinese, which were unlikely to become DSA. A11(A*11:02), A26(A*26:01), A24(A*24:02) and A1(A*01:01)were with high frequencies and the corresponding antigen frequencies were relatively high, especially A24, which was most likely to become a preformed DSA. There were no significant differences among three age groups in the frequencies of anti-HLA-A specificities.

**Table 3:** The frequencies of anti-HLA-A specificities in the three age groups

No	Specificity	Allele	Specificity frequencies (%)			Allele frequency in Chinese (%)
			**Young (*n* = 425)**	**Middle (*n* = 562)**	**Elderly (*n* = 352)**	
1	A80	A*80:01	6.353	4.804	5.114	0.000
2	A68	A*68:02	5.412	5.516	5.966	0.054
3	A11	A*11:02	5.176	3.381	3.977	1.752
4	A34	A*34:01	4.706	3.915	3.977	0.042
5	A43	A*43:01	4.706	3.025	4.261	0.000
6	A26	A*26:01	4.471	3.381	3.693	2.841
7	A66	A*66:02	4.235	4.093	4.830	0.000
8	A66	A*66:01	4.000	3.381	3.409	0.060
9	A2	A*02:03	3.765	3.203	2.557	3.479
10	A24	A*24:02	3.765	5.338	4.261	15.526
11	A29	A*29:01	3.765	3.025	2.841	0.671
12	A29	A*29:02	3.765	3.025	3.409	0.065
13	A1	A*01:01	3.529	3.559	3.409	3.581
14	A23	A*23:01	3.529	5.160	4.261	0.261
15	A24	A*24:03	3.529	5.160	3.693	0.150
16	A25	A*25:01	3.294	4.448	3.125	0.021

*n*:the total number of antibodies at each locus detected in the study

**Table 4:** The frequencies of anti-HLA-B specificities in the three age groups

No	Specificity	Allele	Specificity frequency (%)			Allele frequency in Chinese (%)
			**Young (*n* = 583)**	**Middle (*n* = 1030)**	**Elderly (*n* = 695)**	
**1**	B76	B*15:12	8.405	5.922	6.907	0.231
**2**	B45	B*45:01	6.175	3.592	3.885	0.112
**3**	B57	B*57:01	5.66	3.107	2.878	1.166
**4**	B57	B*57:03	5.146	3.01	3.309	0.005
**5**	B58	B*58:01	4.803	2.816	2.302	6.128
**6**	B37	B*37:01	4.46	2.233	3.166	1.398
**7**	B44	B*44:02	3.945	2.816	3.453	0.87
**8**	B44	B*44:03	3.945	3.107	2.734	2.746
**9**	B67	B*67:01	3.602	2.816	2.878	0.718
**10**	B82	B*82:01	3.602	3.398	3.597	0
**11**	B13	B*13:02	1.887	2.621	2.878	6.268
**12**	B75	B*15:02	1.887	2.621	2.446	3.588

*n*:the total number of antibodies at each locus detected in the study

**Table 5:** The frequencies of anti-HLA-C specificities in the three age groups

No	Specificity	Allele	Specificity frequency (%)			Allele frequency in Chinese (%)
			**Young (*n* = 243)**	**Middle (*n* = 284)**	**Elderly (*n* = 198)**	
**1**	Cw17	C*17:01	19.342	15.141	17.677	0.102
**2**	Cw4	C*04:01	9.054	6.69	8.586	5.769
**3**	Cw18	C*18:02	9.054	7.042	10.101	0.002
**4**	Cw7	C*07:02	7.819	7.747	7.071	15.191
**5**	Cw2	C*02:02	6.584	6.69	9.596	0.69
**6**	Cw5	C*05:01	6.584	6.69	6.566	0.852
**7**	Cw6	C*06:02	6.584	6.69	8.081	8.855
**8**	Cw15	C*15:02	4.938	6.69	7.576	3.347
**9**	Cw10	C*03:02	4.527	4.578	3.535	5.932
**10**	Cw9	C*03:03	4.527	5.986	3.535	6.934
**11**	Cw12	C*12:03	3.704	4.225	4.04	1.93
**12**	Cw16	C*16:01	3.292	4.93	2.02	0.005

*n*:the total number of antibodies at each locus detected in the study

**Table 6:** The frequencies of anti-HLA-DR specificities in the three age groups

No	Specificity	Allele	Specificity frequency (%)			Allele frequency in Chinese (%)
			**Young (*n* = 416)**	**Middle (*n* = 581)**	**Elderly (*n* = 501)**	
**1**	DR51	DRB5*01:01	4.567	2.582	3.393	10.960
**2**	DR4	DRB1*04:02	4.087	3.442	5.190	0.176
**3**	DR1	DRB1*01:02	4.087	2.926	3.393	0.265
**4**	DR52	DRB3*02:02	3.846	3.615	4.591	20.270
**5**	DR4	DRB1*04:04	3.846	4.131	3.792	0.734
**6**	DR10	DRB1*10:01	3.606	2.582	2.595	1.585
**7**	DR18	DRB1*03:02	3.606	3.615	2.994	0.000
**8**	DR4	DRB1*04:01	3.606	3.098	2.794	0.997
**9**	DR9	DRB1*09:01	3.606	2.926	4.790	14.737
**10**	DR9	DRB1*09:02	3.606	2.926	3.792	0.000
**11**	DR12	DRB1*12:01	3.365	3.787	3.792	2.428
**12**	DR4	DRB1*04:03	3.365	3.787	2.595	1.557
**13**	DR14	DRB1*14:01	3.125	4.131	3.792	0.005
**14**	DR53	DRB4*01:01	2.885	3.098	3.792	0.320
**15**	DR8	DRB1*08:01	2.644	3.787	2.794	0.051
**16**	DR7	DRB1*07:01	2.404	3.959	2.794	9.614
**17**	DR14	DRB1*14:54	2.404	3.270	2.794	2.422

*n*:the total number of antibodies at each locus detected in the study

**Table 7:** The frequencies of anti-HLA-DQ specificities in the three age groups

No	Specificity	Allele	Specificity frequency (%)			Allele frequency in Chinese (%)
			**Young (*n* = 348)**	**Middle (*n* = 432)**	**Elderly (*n* = 372)**	
**1**	DQ7	DQA1*03:01 DQB1*03:01	8.046	7.407	8.065	21.086
**2**	DQ8	DQA1*03:01 DQB1*03:02	6.322	4.630	7.527	5.745
**3**	DQ7	DQA1*05:03 DQB1*03:01	6.322	7.407	6.720	21.086
**4**	DQ2	DQA1*05:01 DQB1*02:01	5.172	3.241	5.108	4.935
**5**	DQ6	DQA1*01:03 DQB1*06:03	4.885	3.241	3.763	1.474
**6**	DQ7	DQA1*06:01 DQB1*03:01	4.885	5.787	5.108	21.086
**7**	DQ6	DQA1*01:02 DQB1*06:04	4.598	4.167	4.032	1.436
**8**	DQ4	DQA1*02:01 DQB1*04:01	4.598	3.241	5.645	4.501
**9**	DQ7	DQA1*05:05 DQB1*03:19	4.598	3.472	4.301	0.001
**10**	DQ7	DQA1*02:01 DQB1*03:01	4.310	5.324	5.645	21.086
**11**	DQ8	DQA1*02:01 DQB1*03:02	4.310	4.167	2.688	5.745
**12**	DQ8	DQA1*03:02 DQB1*03:02	4.310	4.630	3.763	5.745
**13**	DQ9	DQA1*03:02 DQB1*03:03	4.023	4.630	3.226	15.884
**14**	DQ9	DQA1*02:01 DQB1*03:03	3.161	4.861	2.151	15.884
**15**	DQ9	DQA1*03:01 DQB1*03:03	3.161	4.167	2.419	15.884
**16**	DQ2	DQA1*04:01 DQB1*02:01	2.874	3.241	4.570	4.935

*n*:the total number of antibodies at each locus detected in the study

**Table 8:** The frequencies of anti-HLA-DP specificities in the three age groups

No	Specificity	Allele	Specificity frequency (%)			Allele frequency in Chinese (%)
			**Young (*n* = 362)**	**Middle (*n* = 403)**	**Elderly (*n* = 429)**	
**1**	DP1	DPA1*02:01 DPB1*01:01	18.508	15.633	13.753	0.120
**2**	DP5	DPA1*02:02 DPB1*05:01	8.564	11.166	8.858	41.920
**3**	DP1	DPA1*01:03 DPB1*01:01	4.696	5.211	3.030	0.120
**4**	DP11	DPA1*01:03 DPB1*11:01	4.144	2.978	4.429	/
**5**	DP19	DPA1*01:03 DPB1*19:01	3.867	3.722	2.098	0.740
**6**	DP11	DPA1*02:02 DPB1*11:01	3.867	3.474	3.263	/
**7**	DP13	DPA1*03:01 DPB1*13:01	3.867	2.978	5.361	7.390
**8**	DP10	DPA1*02:02 DPB1*10:01	3.591	3.474	4.662	0.090
**9**	DP3	DPA1*01:03 DPB1*03:01	3.315	2.978	3.730	4.770
**10**	DP6	DPA1*01:03 DPB1*06:01	3.039	2.233	3.263	/
**11**	DP20	DPA1*03:01 DPB1*20:01	3.039	3.474	4.895	/
**12**	DP28	DPA1*04:01 DPB1*28:01	3.039	3.226	3.030	0.300
**13**	DP14	DPA1*02:01 DPB1*14:01	2.762	3.970	2.098	3.690
**14**	DP15	DPA1*02:01 DPB1*15:01	2.762	2.481	3.730	0.120
**15**	DP28	DPA1*01:05 DPB1*28:01	2.486	2.978	2.797	0.300

*n*:the total number of antibodies at each locus detected in the study

**Table 9:** The frequencies of anti-MICA specificities in the three age groups

No	Specificity	Allele	Specificity frequency (%)			Allele frequency in Chinese (%)
			**Young (*n* = 129)**	**Middle (*n* = 111)**	**Elderly (*n* = 133)**	
**1**	MICA07	MICA*007	12.403	11.712	9.774	1.250
**2**	MICA02	MICA*002	10.853	9.009	9.023	14.750
**3**	MICA12	MICA*012	10.078	13.514	10.526	4.750
**4**	MICA01	MICA*001	9.302	15.315	8.271	/
**5**	MICA17	MICA*017	6.977	5.405	3.008	0.000
**6**	MICA06	MICA*006	6.202	6.306	9.023	/
**7**	MICA09	MICA*009	6.202	3.604	9.023	10.000
**8**	MICA28	MICA*028	6.202	6.306	7.519	0.250
**9**	MICA04	MICA*004	5.426	3.604	7.519	3.500
**10**	MICA15	MICA*015	5.426	6.306	3.759	0.000
**11**	MICA46	MICA*046	5.426	5.405	1.504	/
**12**	MICA05	MICA*005	4.651	4.505	8.271	/
**13**	MICA18	MICA*018	3.101	5.405	2.256	0.250
**14**	MICA27	MICA*027	3.101	1.802	6.767	6.750

*n*:the total number of antibodies at each locus detected in the study

The top two frequent anti-HLA-B specificities in the three age groups were B76(B*15:12)and B45(B*45:01). Especially, the frequency of B76 was very prominent, but it was unlikely to become a DSA in East China for the corresponding antigen frequency was not common in Chinese. B57(B*57:01),B58(B*58:01),B44(B*44:03) and B13(B*13:02)were with high frequencies and the corresponding antigen frequencies were also relatively high, which made them prone to be performed DSA. There were no significant differences among three age groups in the frequencies of anti-HLA-B specificities, except significant differences at B45(young vs middle, *P* = 0.0165),B57 (B*57:01,young vs middle, *P* = 0.0122;young vs elderly, *P* = 0.0129),B57(B*57:03,young vs middle, *P* = 0.031),B58(young vs middle, *P* = 0.038,young vs elderly, *P* = 0.0146),B37(young vs middle, *P* = 0.0123).

The most frequent anti-HLA-C specificity in the three age groups was C17 and its frequency was more than twice of the second ranked antibody. While the corresponding antigen frequency was low in Chinese, so it was unlikely to become a DSA in East China. Cw4(C*04:01), Cw7(C*07:02), Cw6(C*06:02), Cw15(C*15:02), Cw10(C*03:02), Cw9(C*03:03), Cw12(C*12:03) would more likely to become preformed DSA. There were no significant differences among three age groups in the frequencies of anti-HLA-C specificities.

Anti-HLA-DR specificities were evenly distributed in every age group and DR9 (DRB1*09:01) and DR7 (DRB1*07:01) were the most two likely to become DSA. In addition, DR12 (DRB1*12:01), DR4 (DRB1*04:03), DR14 (DRB1 *14:54) and DR10 (DRB1*10:01) were more likely to become DSA. There were no significant differences among the three age groups in the frequencies of anti-HLA-DR specificities.

At HLA-DQ locus, the antibody specificities with high frequencies were coincided with the alleles with high frequencies in Chinese, which made them more likely to become preformed DSA compared with other loci. DQ7(DQB1*03:01),DQ8(DQB1*03:02),DQ9(DQB1*03:03),DQ2(DQB1*02:01), DQ6(DQB1*06:03),DQ6(DQB1*06:04) and DQ4(DQB1*04:01) were more likely to become potential DSA. There were no significant differences among three age groups in the frequencies of anti-HLA-DQ specificities, except frequency of DQ9(DQA1*02:01 /DQB1*03:03) significant higher in the middle than in elderly group (*P* = 0.0399).

The most frequent anti-HLA-DP specificities in the three age groups was DP1(DPB1*01:01),but the corresponding antigen was not common in Chinese, which made it unlikely to become a DSA. DP5 (DPB1*05:01), DP13 (DPB1*13:01), DP3 (DPB1*03:01) and DP14 (DPB1*14:01) were more likely to become potential DSA. There were no significant differences among three age groups in the frequencies of anti-HLA-DP specificities.

There were no significant differences at anti-MICA specificities among the three age groups. MICA07, MICA02, MICA09, MICA12 were more likely to become potential DSA.

## Discussion

With the advances in supportivecare, better donor selection and the development of reduced intensity conditioning (RIC) extended the indications for allogeneic HSCT to wider patients with hematologic malignancies including elderly one with more emphasis on comorbidities than age alone for patient selection [13, 14]. Ageing affected humoral immune response both quantitatively and qualitatively, as specificity and class of antibody produced were changed and immunosenescence in individuals generally began at the age over 50 years old [15, 16]. Preformed anti-HLA and anti-MICA antibodies in recipients were of great importance to the transplant outcomes of HLA-mismatched allogeneic HSCT and Ciurea et al. found that DSA was the only risk factor for graft failure [17].However, there was a lack of research focusing on age-associated changes on performed anti-HLA and anti-MICA antibodies in recipients prior to HLA-mismatched allogeneic HSCT.

The objective of this study was to try to evaluate ageing on the impact of distribution about preformed anti-HLA and anti-MICA antibody specificities in recipients prior to initial HSCT. In this study, we found that there were nearly no differences in the distribution of anti-HLA and anti-MICA antibodies in different age groups before HSCT, except that a significant higher prevalence of anti-HLA class I and class II antibodies in the elderly than in young group. As age advanced, the more exogenous antigen stimulation, the higher the positive rate of anti-HLA antibodies, which was consistent with the immunity rhythm. Notably, once anti-HLA antibodies occurred, they were not easily to be eliminated. Despite the ambiguity due to differences between techniques and laboratories, the overall consensus was that DSA level with MFI > 5000 was the significant risk factor for recipients receiving HLA- mismatched HSCT [18]. In this study we found there were no differences in the distribution of anti-HLA antibodies among three age groups at maximum MFI > 5000. So we speculated that the risk of preformed anti-HLA antibodies for development of graft failure in different age groups was basically same. Besides, we observed that the prevalence of preformed anti-HLA class I antibodies was significantly higher than class II, and class II than MICA in the young group. This pattern was consistent with the distribution of natural antibodies. Natural antibodies were obtained from young males with the mean age of 33.1 + /_10.3 years [19]. This also indicated that anti-HLA class I antibodies were more prone to be generated in the early years. The higher prevalence of anti-HLA class I antibodies than that of class II antibodies can be ascribed to the fact that all nucleated cells express anti-HLA class I antigens, while class II antigens are only found in a subgroup of immune cells [20]. Besides, platelet (PLT) transfusion may lead to anti-HLA class I antibodies.

In this study, we found the frequencies of specificities against HLA-A, -B, -C, -DR, -DQ, -DP and MICA antigens in the three age groups were generally consistent. There were no significant differences in frequencies of anti-HLA-A,-C,-DR,-DP and MICA specificities among the three age groups, except minority specificities of anti-HLA-B,-DQ antibodies with significant differences. The frequencies and specificities of anti-HLA class I antibodies in the three age groups were very similar and coincided to that of the natural HLA antibodies found in healthy people. Natural anti-HLA antibodies emerged from cross-reactions with common environmental non-self HLA antigens encountered all along their lives [21]_,_ which was unlikely to produce hyperacute rejections. We observed that the common preformed anti-HLA class I specificities were against rare antigens such as A80,A68, B76, B45,Cw17. At HLA-A locus, the most two frequent specificities in the three age groups were A80 (A*80:01) and A68(A*68:02). A*80:01 was an old allele of African origin and A*68:02 was a common allele in Sub-Saharan Africa Black [22, 23]. Fortunately, this type of sensitization seemed to be very specific to less frequent antigens in Chinese, which meant donors carrying these types of HLA antigens would be easily avoided. At HLA-B locus, the most two frequent specificities in the three age groups were B76 and B45. As a "short" antigen, B76 was defined by a loss of serological reactivity. Different substitutions in the same region of the a2 helix may well lead to loss of the same epitopes and they could give rise to similar gain of B45 cross-reactive epitopes [24]. That was the reason B76 and B45 were both very frequent at the same time. At HLA-C locus, the most frequent specificity in the three age groups was Cw17 and the corresponding antigen was found with high frequency in populations of African descent [25]. Cw17 was a most unusual molecule defining a third allelic lineage at this locus. Although the SAFB assay demonstrated high sensitivity and specificity for detecting anti-HLA antibodies, the occurrence of false-positive reactivity was inescapable. That was the reactivity was not representative of genuine HLA antibody and which had no effect on clinical outcomes including graft rejection and survival [26–28]. As far as we know, anti-HLA A80,B76,B45,Cw17 were known to be famous false positives [29, 30]. In this study, we observed that the common preformed anti-HLA class II specificities were against common antigens such as DR9, DR7, DR12, DR4, DR14, DR10; DQ7, DQ8, DQ9, DQ2, DQ6, DQ4; DP1, DP5, DP13, DP3, DP14.At HLA-DR locus, DRB1* 09:01 and DRB1* 07:01 were the most two frequent alleles in Chinese, especially in Jiangsu population [31]. HLA-DQB1*03:01 was the most frequent allele in Chinese and Jiangsu population at HLA-DQ locus and DQB1*03:02, DQB1*03:03,DQB1*02:01,DQB1*06:03,DQB1*06:04, DQB1*04:01 were also frequent in Chinese. So anti-HLA class II antibodies were most likely to become potential DSA, especially anti-HLA-DQ antibodies. Several factors may explain the prominence of DQ antibodies, such as greater potential peptide variability, and a high degree of self-chain paired with non-self epitope [32]. In recent years, more and more studies confirmed that non-permissive HLA-DPB1 mismatching and the presence of performed DSA with the donor HLA-DPB1-mismatched antigens were associated with increased risk of graft failure in HSCT [33, 34]. Anti-HLA-DP1 antibody was with a frequency of 20.5% in this study. DPB1*01:01 was a common allele in Sub-Saharan Africa Black while a rare allele in Chinese. Besides anti-HLA DP1 was a famous false positive antibody [29, 30].

In contrast to solid organ transplant, anti-HLA antibodies in HSCT represent a unique challenge in that the source of those antibodies, the host immune system, is replaced by the process of transplantation. In case of DSA and in the absence of an alternative donor, reduction of anti-HLA antibodies through desensitization treatments can limit the risk of graft failure and facilitate engraftment. Direct antibody removal with therapeutic plasma exchange (TPE) has been used, and chemotherapeutics or monoclonal antibodies, such as bortezomib and rituximab, targeting antibody production have been used [1].

Preformed anti-MICA antibodies were found to correlate with low levels of sMICA after transplantation and consequently with a low incidence of GVHD, which was explained by neutralizing effect of anti-MICA antibodies on sMICA. In this study, we observed the prevalence of anti-MICA antibodies was increased associated with ageing and there was a higher significant difference of positive rate of maximum MFI > 5000 in the elderly than in young group, which might induce a better HSCT transplantation effect in the elderly group.

## Conclusions

In conclusion, the prevalence of anti-HLA antibodies increased slightly associated with ageing, while the risk for developing preformed anti-HLA antibodies resulting in graft failure in HSCT was not increased. Ageing had a small impact on the distribution of antibody specificities against HLA-A, -B, -C, -DR,-DQ, -DP and MICA antigens in recipients awaiting initial HSCT in this study. The distribution of preformed anti-HLA class I antibodies was similar to that of the natural antibodies. Anti-HLA class II antibodies were more likely to become DSA than class I. DR7, DR9, DQ7, DQ8, DQ9 were more likely to become DSA in East China. On the contrary, the prevalence rate of anti-MICA antibodies increased associated with ageing, which might be beneficial for the elderly in HLA-mismatched HSCT.

There were several limitations in this study. First, it was a single center study and this study was limited to Chinese populations. Second, about the ageing question, it would be more meaningful to show longitudinal data regarding the evolution of anti-HLA antibodies with age rather than one single time point.

## Methods

### Subjects

Data on 426 unrelated Han ethnic patients with hematologic malignancies awaiting initial HSCT in the First Affiliated Hospital with Nanjing Medical University of East China from November 2016 to April 2023 were collected retrospectively in this study. HLA-matching was based on high resolution typing for HLA-A,B,C,DRB1 and -DQB1.Haploidentical related donor (at least 5/10) and 9/10 matched unrelated donor were criteria of HLA-mismatched donors in this study with the donor age from 15 to 55.All serum samples were taken and detected for anti-HLA class I, class II and anti-MICA antibodies within 2 weeks prior to HSCT. General clinical characteristics were obtained from medical history in the hospital. Transfusions including PLT and leukoreduced RBC transfusions three times or more and pregnancy once or more were involved in this study. The patients were stratified into three groups based on age: young (≤ 30 years), middle (31 -50 years) and elderly (≥ 51 years). In this retrospective study, ethical approval was obtained by the ethics commit of the First Affiliated Hospital with Nanjing Medical University (2023-SR-463) with exemption from informed consent for patients.

### Detection of anti-HLA and anti-MICA antibodies

All serum samples were detected with SAFB assay for anti-HLA class I, class II and anti-MICA antibodies within 2 weeks prior to HSCT using LABScreen single antigen (LS1A04, LS2A01, LSMICA001, One Lambda, Canoga Park, CA) according to the manufacturer’s protocol. The fluorescent signal of antigen specific coated bead was detected using LABScan 3D Flow Cytometry (Luminex Inc., Austin, TX) and analyzed by Luminex® xPONENT. Antibody specificity and binding level were analyzed and determined through HLA Fusion 4.6.0 software. LABScreen single antigen results were reported as molecular specificities with a positive cutoff value of 500 MFI in our laboratory. All specificities and MFI’s were exported for statistical analysis.

### Statistical analysis

The negative ratio, positive rate of anti-HLA class I, class II and anti-MICA antibodies at maximum MFI of 500–3000(weak), 3000–5000(intermediate), 5000–10000(strong), > 10,000(very strong) were compared and analyzed among the three age groups. The specificities with MFI > 500 were listed according to HLA-A, -B, -C,-DR,-DP,-DQ and MICA loci separately. The antibody specificity frequencies against HLA-A,-B,-C,-DR,-DP,-DQ and MICA antigens were determined using the number of each specificity dividing the total number of antibodies at each locus detected in the study. The antibody specificity frequencies were compared and analyzed among the three age groups (compared between every two different age groups.) The Statistical Package for Social Sciences software (version 17.0, SPSS Inc., Chicago, IL, USA) was used to conduct statistical analysis for every two different age groups. The chi-square test was used to compare the negative ratio, positive rate, antibody specificity frequencies against HLA-A, -B, -C,-DR,-DP,-DQ and MICA antigens. A *P*-value < 0.05 was considered statistically significant.

## Data Availability

The data that support the findings of this study are available from the corresponding author upon reasonable request.

## Abbreviations

HSCT: Hematopoietic Stem Cell Transplantation
HLA: Human leukocyte antigen
DSA: Donor-specific HLA antibodies
MICA: MHC class I chain related antigens A
GVHD: Graft-versus-host disease
SAFB: Single antigen flow bead
MFI: Mean fluorescent intensity
AML: Acute myeloid leukemia
ALL: Acute lymphoblastic leukemia
MDS: Myelodysplastic syndrome
CMML: Chronic myelomonocytic leukemia
AA: Aplastic anemia
RIC: Reduced intensity conditioning
PLT: Platelet
TPE: Therapeutic plasma exchange
