# Correction: The impact of ageing on the distribution of preformed anti-HLA and anti-MICA antibody specificities in recipients from eastern China prior to initial HSCT

**DOI:** 10.1186/s12979-024-00428-1

**Published:** 2024-04-13

**Authors:** Qinqin Pan, Xiao Ma, Yajie You, Yuejiao Yu, Su Fan, Xiaoyan Wang, Mengyuan Wang, Ming Gao, Guangming Gong, Kourong Miao, Jie Shen, Xiaoyu Zhou

**Affiliations:** 1 HLA Lab, Department of Transfusion, The First Affiliated Hospital of Nanjing Medical University, Jiangsu Province Hospital, Nanjing, 210029 China; 2 Department of Pharmacy, Jinling Hospital, Nanjing University School of Medicine, Nanjing, 210002 China; 3 Department of Hematology, The First Affiliated Hospital of Nanjing Medical University, Jiangsu Province Hospital, Nanjing, 210029 China

**Correction: Immun Ageing 21, 15 (2024)**.

10.1186/s12979-024-00417-4

Following publication of the original article [1], the Editors-in-Chief of *Immunity & Ageing* requested to update the article title with the approval of the authors from “Ageing on the impact of distribution about preformed anti‑HLA and anti‑MICA antibody specificities in recipients prior to initial HSCT from East China” to “The impact of ageing on the distribution of preformed anti-HLA and anti-MICA antibody specificities in recipients from eastern China prior to initial HSCT’.

The original article [1] has been updated.
